# Genetic and epigenetic characterization of sarcoma stem cells across subtypes identifies EZH2 as a therapeutic target

**DOI:** 10.1038/s41698-024-00776-7

**Published:** 2025-01-09

**Authors:** Edmond O’Donnell, Maria Muñoz, Ryan Davis, Jessica Bergonio, R. Lor Randall, Clifford Tepper, Janai Carr-Ascher

**Affiliations:** 1 Department of Orthopedic Surgery, University of California Davis, Sacramento, CA 95817 USA; 2 Department of Internal Medicine, Division of Hematology/Oncology, University of California Davis, Sacramento, CA 95817 USA; 3 Department of Pathology and Laboratory, University of California Davis, Sacramento, CA 95817 USA

**Keywords:** Sarcoma, Cancer stem cells

## Abstract

High-grade soft tissue sarcomas (STS) are a heterogeneous and aggressive set of cancers. Failure to respond anthracycline chemotherapy, standard first-line treatment, is associated with poor outcomes. We investigated the contribution of STS cancer stem cells (STS-CSCs) to doxorubicin resistance. We identified a positive correlation between CSC abundance and doxorubicin IC_50_. Utilizing patient-derived samples from five sarcoma subtypes we investigated if a common genetic signature across STS-CSCs could be targeted. We identified Enhancer of Zeste homolog 2 (EZH2), a member of the polycomb repressive complex 2 (PRC2) responsible for H3K27 methylation as being enriched in CSCs. EZH2 activity and a shared epigenetic profile was observed across subtypes and targeting of EZH2 ablated the STS-CSC population. Treatment of doxorubicin-resistant cell lines with tazemetostat resulted in a decrease in the STS-CSC population. These data confirm the presence of shared genetic programs across distinct subtypes of CSC-STS that can be therapeutically targeted.

## Introduction

High-grade complex karyotype sarcomas are treated with aggressive multimodality treatment including surgery, radiation, and chemotherapy. Despite this, the prognosis for these patients is poor with a 65% five-year survival rate in patients that present with localized disease^1^. This is due to the failure of primary therapy leading to recurrence and metastatic disease. The development of new treatments is complicated by the heterogeneity of disease, evidenced by the more than 70 sarcoma subtypes with varying histology, genetics, and patient demographics^1,2^. Sarcomas are classified into two genetic groups, simple or complex karyotype. Simple karyotype sarcomas have a known translocation, such as SYT::SSX that drives synovial sarcoma^3^. Complex karyotype sarcomas display an aneuploidy phenotype with numerous chromosomal gains and losses^4^. In adults, over 85% of sarcomas have a complex karyotype including undifferentiated pleomorphic sarcoma (UPS), the most common sarcoma subtype. These tumors contain a vast number of chromosomal changes; however, there is a lack of common critical regulatory pathways underlying this aggressive disease^2,3^. Identifying new therapeutic targets across subtypes will result in improved patient outcomes.

Sarcoma mortality is primarily driven by treatment failure and development of metastatic disease. Cancer stem cells (CSCs) are a subpopulation of cells within the bulk tumor that can initiate tumor formation and grow indefinitely^5^. This population has stem cell-like properties, including protection from apoptosis resulting in therapy resistance and increased metastatic potential^5^. Therefore, there is considerable interest in identifying therapeutic vulnerabilities in sarcoma CSCs as this population is contributing to patient mortality. The majority of patients with high-risk localized disease or metastatic sarcoma are treated with doxorubicin, an anthracycline-based chemotherapy. Resistance to doxorubicin in sarcoma has been attributed to upregulation of multidrug resistance (MDR) efflux pumps and increased expression of anti-apoptotic factors such as BCL-2^6,7^. Sarcoma CSCs have been identified across several subtypes such as rhabdomyosarcoma and malignant peripheral nerve sheath tumor (MPNST)^8,9^. Within these sarcomas, CSCs have upregulation of hedgehog, notch, and hippo signaling. In rhabdomyosarcoma, a childhood skeletal muscle tumor, YAP has been shown to promote and maintain stem cells along with NOTCH and SOX^10,11^. An evaluation of CSCs across common complex karyotype sarcomas to identify shared pathways and opportunities for therapeutic targeting has not been performed.

In this study, we observed that doxorubicin resistance in soft tissue sarcomas is associated with an increase in CSC abundance. We hypothesized that a common genetic signature could be identified across CSCs and targeted to overcome resistance. To evaluate this, we performed the first genetic evaluation of sarcoma CSCs across subtypes. From five common sarcoma subtypes, we identified EZH2, a member of the PRC2 epigenetic complex, as being upregulated across the disease spectrum. Targeting of EZH2 led to a reduction of the CSC population and sensitization to doxorubicin indicating this as a potential adjuvant therapeutic strategy for an aggressive and treatment-resistant disease.

## Results

### Acquired resistance to anthracycline chemotherapy correlates with an increase in cancer stem cells

**Fig. 1:**
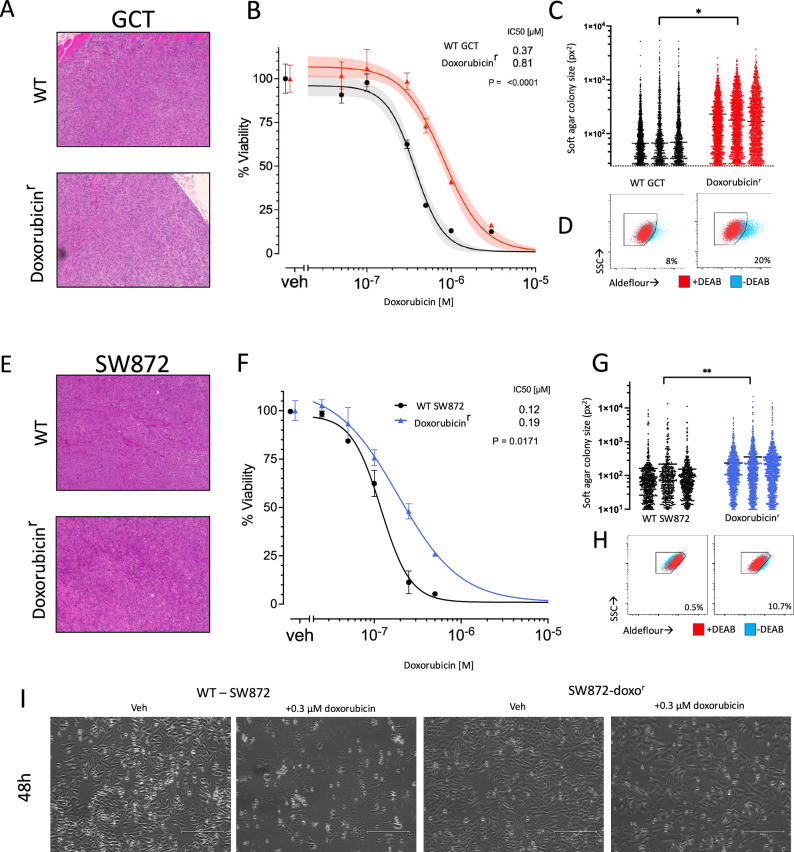
Acquired doxorubicin resistance correlates with increased soft tissue sarcoma cancer stem cell abundance and phenotype. **A** A doxorubicin-resistant (doxo^R^) derivative line of WT GCT (undifferentiated pleomorphic sarcoma) cells was generated through serial passage in increasing concentrations of doxorubicin. H&E stains of representative subcutaneous tumors generated from WT and Doxo^R^ SW872 cells. **B** H&E stains of representative subcutaneous tumors generated from WT and Doxo^R^ cells SW872 cells (dedifferentiated liposarcoma). **C** Cell-titer glo viability assays for doxorubicin dose-response in WT and doxoR derivative GCT cells. Data points are the mean ± SD of triplicate wells and are representative of three different experiments. **D** A doxorubicin-resistant (doxo^R^) derivative line of WT SW872 cells was generated through serial passage in doxorubicin and exhibited an increased IC_50._ Data points are the mean ± SD of triplicate wells and are representative of three different experiments. **E** Representative photographs of SW872 WT and Doxo^R^ cells after 48 hours of treatment with either DMSO (vehicle) or 300 nM doxorubicin. bars = 250 nm. **F** WT and Doxo^R^ GCT cells were subjected to soft-colony formation assays and the abundance and size of colonies was counted with imageJ software. Each column is a biological replicate. Colony size is shown in units as pixels (px^2^). **G** Aldefluor assay showing increased bright population in doxo^R^ (right) cells vs WT GCT cells (left). The DEAB controls are shown in red (*n* = 3). **H** WT and Doxo^R^ SW872 cells were subjected to soft-colony formation assays and the abundance and size of colonies was counted with imageJ software. Colony size is shown in arbitrary units as pixels^2^. **I** Aldefluor assay showing increased bright population in doxo^R^ (right) cells vs WT SW872 cells (left) (*n* = 3). *P < 0.05, **P < 0.05.

Doxorubicin, an anthracycline-based chemotherapy is the cornerstone of systemic treatment across high-grade complex karyotype sarcoma subtypes. While this is effective, acquired resistance can occur resulting in recurrent or metastatic disease. Resistance to anthracycline therapy is associated with poor prognosis^12^. Across tumor types, literature suggests that therapeutic resistance and metastasis are attributable to the cancer stem cell population^5^. To investigate this in sarcoma, we created doxorubicin-resistant cell lines. We cultured GCT, a cell line representing undifferentiated pleomorphic sarcoma (UPS) and SW872 derived from dedifferentiated liposarcoma (LPS) with increasing concentrations of doxorubicin (0.05–0.4 uM) over the course of four months. Histologically, xenograft tumors formed from these cell lines were indistinguishable from the parental line (Fig. 1A, B). Resistance to doxorubicin (Doxo^R^) was verified with dose-response curves. The doxorubicin-resistant GCT (GCT-Doxo^R^) cells had a significantly higher IC50 value as compared to wild-type cells (Fig. 1C, D). This was also observed in doxorubicin-resistant SW872 (SW872-Doxo^R^) cells as compared to WT SW872 lines (Fig. 1D, E). Importantly, the resistance to doxorubicin was stable in culture over several months in the absence of treatment.

To investigate the relationship between doxorubicin resistance and cancer stem cells, we evaluated functional differences in resistant and wild-type cell lines. We first subjected WT and Doxo^R^ cells to soft agar assay given that the ability to grow in anchorage-independent conditions is associated with a CSC phenotype. We used image-based analysis to quantify the size and abundance of colonies (Fig. 1F, H). In both the GCT and SW872 cell model systems, colonies generated by Doxo^R^ derivatives were both more numerous per assay as well as larger in size. This result suggested a more abundant population of cancer stem cells within Doxo^R^ derivative lines compared to the parent cell line following the acquisition of chemoresistance. To further evaluate this possibility, we subjected these cell models to the Aldefluor assay, a well-established technique to measure the aldehyde dehydrogenase activity of cells. Consistent with a CSC phenotype, there was an increased abundance of Aldefluor bright cells in the SW872 and GCT Doxo^R^ cells (Fig. 1G, I).

### Genetic characterization of cancer stem cells across high-grade complex karyotype sarcomas

**Fig. 2:**
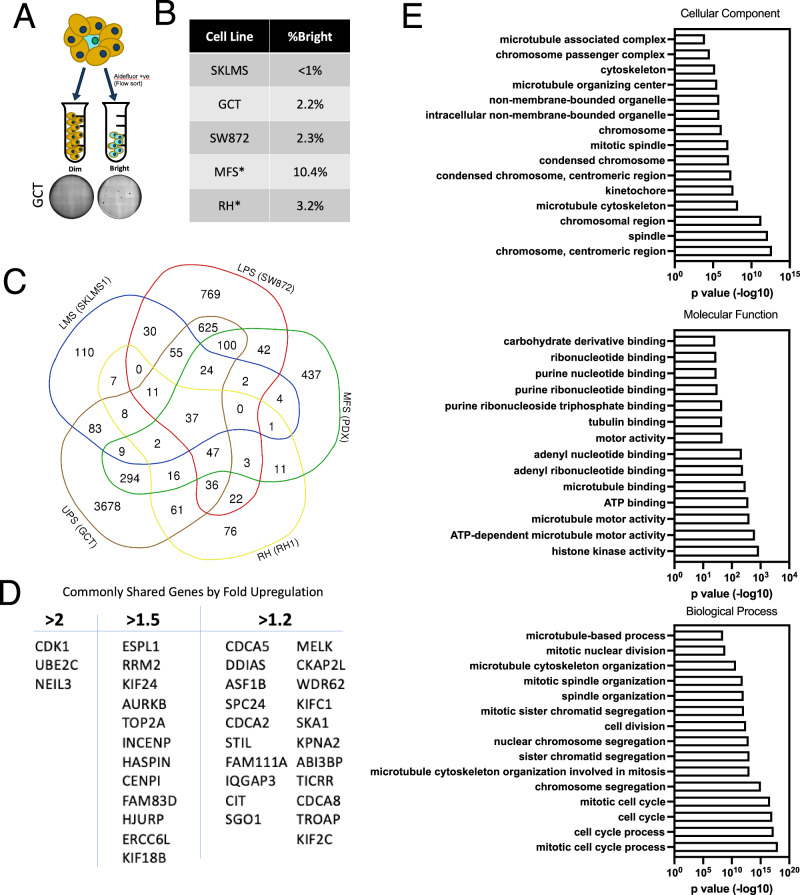
Isolation and RNA-sequencing-based analysis of soft-tissue sarcoma cancer stem cells with Aldefluor sorting reveals a shared gene signature. **A** Sorting scheme of Aldefluor bright and dim sorted cells among five soft tissue sarcoma cell lines. A representative soft-agar colony forming assay from sorted GCT cells shows the increased propensity for anchorage-independent growth in Aldefluor bright cells. **B** Table showing the percent Aldefluor positive population among the five different cell lines. LMS=leiomyosarcoma (SKLMS1), RH=rhabdomyosarcoma, MFS=myxofibrosarcoma, UPS=undifferentiated pleomorphic sarcoma (GCT), LPS=dedifferentiated liposarcoma (SW872). **B** Percent Aldefluor positive (bright) cells for each of the different lines tested. **C** Venn diagram showing the overlap of differentially expressed genes meeting a significance of *p* < 0.05. 36 genes were upregulated and 1 gene (BRCA1) was downregulated. **D** Table of the commonly upregulated genes shown in panel **C** subdivided according to fold change in expression. **E** Gene ontology analysis of the 37 shared, differentially regulated genes across the five sarcoma types.

Acquired resistance to doxorubicin is associated with increased mortality from high-grade sarcomas and we observed this is associated with enrichment of the CSC population. To identify a common target within the CSC population across a diverse subset of sarcomas, we utilized differential gene expression to compare the CSC population to bulk tumor cells. Within sarcomas, there is considerable subtype heterogeneity and a uniform marker of the CSC population is unknown. Across tumor types, aldehyde dehydrogenase (ALDH) is known to be upregulated within CSCs and this has been shown in sarcoma as well. Therefore, we utilized the Aldefluor assay to isolate CSCs from the non-CSC population using cell sorting. To confirm the difference in phenotype, GCT cells were isolated by sorting bright vs. dim cells in the Aldefluor assay, and then subjected to soft-agar formation assays. Aldefluor bright GCT cells exhibited better anchorage-independent growth as compared to the Aldefluor dim non-CSC cells (Fig. 2A).

We selected five high-grade complex karyotype sarcoma cell lines that each represent the most common subtypes in adults. Specifically, leiomyosarcoma, rhabdomyosarcoma, myxofibrosarcoma, undifferentiated pleomorphic sarcoma, and dedifferentiated liposarcoma. Consistent with prior studies, Aldefluor analysis of these cells demonstrated a small number of cells composing the CSC population (Fig. 2B).

Aldefluor bright and dim cells from each subtype were sorted and analyzed by RNA sequencing. Differential gene expression showed significant differences when comparing across subtypes but, interestingly, comparisons of Aldefluor bright to dim cells within sarcoma subtypes demonstrated minimal differences (Supplementary Figure 1). To identify differentially regulated genes across bright and dim populations, we utilized a venn-diagram approach of genes significantly upregulated or downregulated at varying fold expression levels. Of note, only one commonly downregulated gene, BRCA, was identified. (Fig. 2C). A total of 36 genes were differentially upregulated at an FDR < 0.05, comprising 3, 12, and 21 genes by >2, >1.5, and >1.2 fold, respectively (Fig. 2D). The significant differentially expressed genes (*n* = 37) were further evaluated by gene ontology analysis (Fig. 2E). We noted significant enrichment for ontology terms related to the maintenance and integrity of cellular mitosis.

### Enhancer of Zeste Homolog 2 (EZH2) is increased in high-grade complex karyotype sarcoma cancer stem cells

**Fig. 3:**
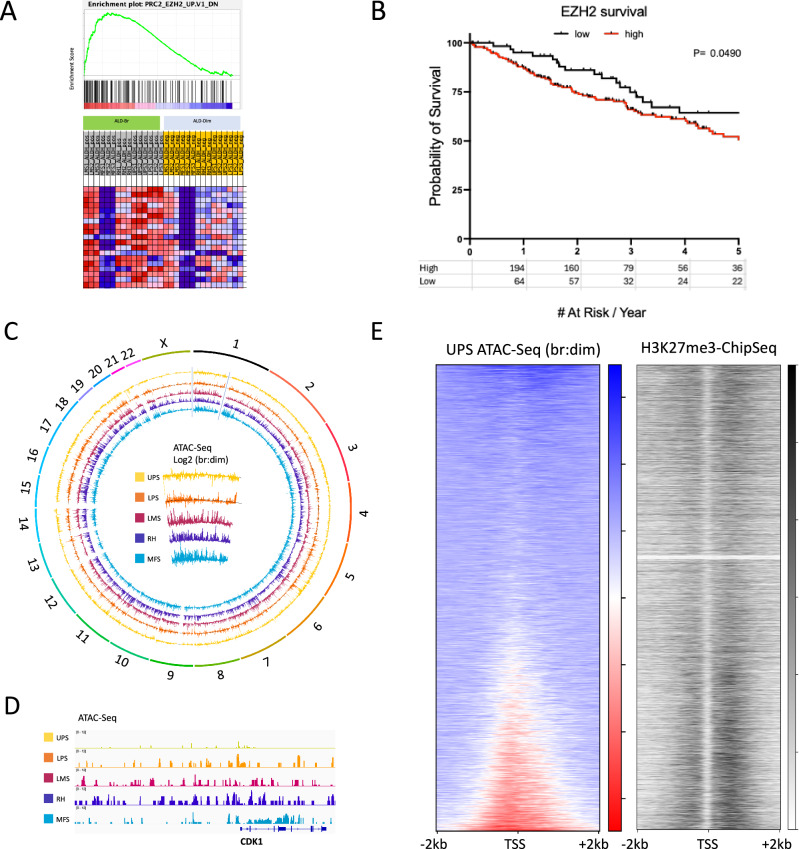
Identification of a shared EZH2 signature among soft tissue sarcoma cancer stem cells. **A** Expression data from RNA-Seq was analyzed by gene set enrichment analysis, and enrichment plot of one of the top-scoring hits is shown along with generative heatmap. **B** Survival analysis (oncolnc.org) showing overall survival among available sarcoma cases within the TCGA database, low expression represents the lowest quartile, while highest expression represents a composite of the three higher quartiles, Log-rank *P* = 0.05. **C** Circos plot of genome wide ATAC-seq data generated from sorted soft-tissue sarcoma bright and dim cells. More ATAC-seq signal (accessible chromatin) in bright vs dim cells directs outward, while decreased chromatin access in bright vs dim cells directs inward. The center plot shows an enlargement of the track data from chromosome 1. **D** ATAC-seq data from the promoter region of CDK1, one of the differentially identified genes by RNA-seq. The window region is 50 kb. **E** Increased chromatin accessibility at transcription start sites (TSS) correlating with the molecular target of EZH2. The plot on the right shows ATAC-seq signal as a ratio of bright to dim in WT GCT (UPS) cells was plotted for all canonical TSS in a 4 kb window, and ordered from low (top) to high (bottom). The plot on the left shows H3K27-me3 Chip-Seq data from a publicly available HepG2 dataset plotted according to the same rank-order as the right plot. Increasing black represents more Chip-Seq signal.

To identify potentially targetable pathways in the CSC population as compared to the non-CSC population, we performed gene set enrichment analysis (GSEA) of differentially expressed genes. From this, we identified enhancer of zeste homolog 2 (EZH2), the catalytic subunit of the polycomb repressive complex 2 (PRC2) that facilitates H3K27 methylation as an enriched pathway (Fig. 3A). However, EZH2 itself was not among the differentially regulated genes in dim versus bright cells (Fig. 2).

As an epigenetic modulator, increased EZH2 expression and activity leads to increased H3K27 methylation and subsequent gene silencing^13^. It has been demonstrated that EZH2 overexpression leads to suppression of differentiation or alternate cell fate pathways^14^. Consistent with this, it has been shown to maintain stem cell populations in both cancer and non-cancer tissues. EZH2 inhibition is an effective therapeutic strategy in epithelioid sarcomas that have a loss of SMARCB1, a subunit of SWI/SNF complex that opposes the activity of EZH2^15^. Adding further support to the clinical relevance of EZH2, we evaluated EZH2 expression with respect to overall survival within The Cancer Genome Atlas (TCGA) dataset, which showed that patients with the highest quartile of EZH2 expression was associated with significantly worse survival compared to sarcomas with the lowest quartile of EZH2 expression (Fig. 3B, *p* = 0.049)^16^. TCGA evaluated seven soft tissue sarcoma subtypes, four that overlap with the subtypes that are the focus of this study (UPS, MFS, LPS, and LMS). These data support further investigations of targeting EZH2 across sarcoma subtypes.

EZH2 modulates chromatin compaction through histone modification and therefore, we evaluated this in the cancer stem cell population using the Assay of Transposase Accessible Chromatin (ATAC-seq), which allows for an assessment of chromatin accessibility^13,17^. In parallel with the Aldefluor-based sorting described above for RNA-Seq analysis, we also subjected the sorted material to ATAC-seq to evaluate differences in chromatin accessibility between bulk cells and CSCs. Following peak-calling and annotation, we noted a general trend in the cell lines tested for an increase in the number of total peaks, although on a percent basis there was little difference among different genetic regions between bright and dim cells (Supplementary Fig. 2). This observation was further visualized using a circos plot of the fold differences between bright and dim cells representing CSCs and non-CSCs (Fig. 3C). On a global level, and consistent with Fig. 3C, there was higher ATAC-Seq signal in bright cells compared to dim cells. We also investigated the ATAC-seq data focused on some of the differentially regulated genes between bright and dim cells. As an example, bright cells had an increased ration of ATAC-seq signal in the promoter of CDK1 compared to dim cells amongst all five the sarcoma lines tested (Fig. 3D). The differences in chromatin accessibility were similar across sarcoma subtypes indicating a shared CSC-specific program.

Having identified shared differential chromatin accessibility within CSCs, we next asked whether there was evidence for an EZH2-specific signature. To address this, we evaluated chromatin accessibility at transcription start sites in an unbiased manner across the genome and compared this with publicly available H3K27-me3 Chip-Seq data. We hypothesized that if EZH2 was involved in regulating chromatin accessibility in these areas, our ATAC-seq data would correlate with an increased H2K27-me3 chip-seq signal. We first ordered our gene list from a high to low ratio of ATAC-seq signal at TSSs, correlating to an ‘open’ vs ‘closed’ or ‘bound’ status, respectively. This ordered set of genes was then plotted against the H3K27me3 signal from a publicly available HepG2 dataset, as a sarcoma-specific dataset was not available. Consistent with our hypothesis, we observed increased H3K27-me3 chip-seq signal was increased in CSCs as compared to the non-CSC population (Fig. 3E).

### Tazemetostat, a small molecule inhibitor of EZH2 abrogates the CSC phenotype

**Fig. 4:**
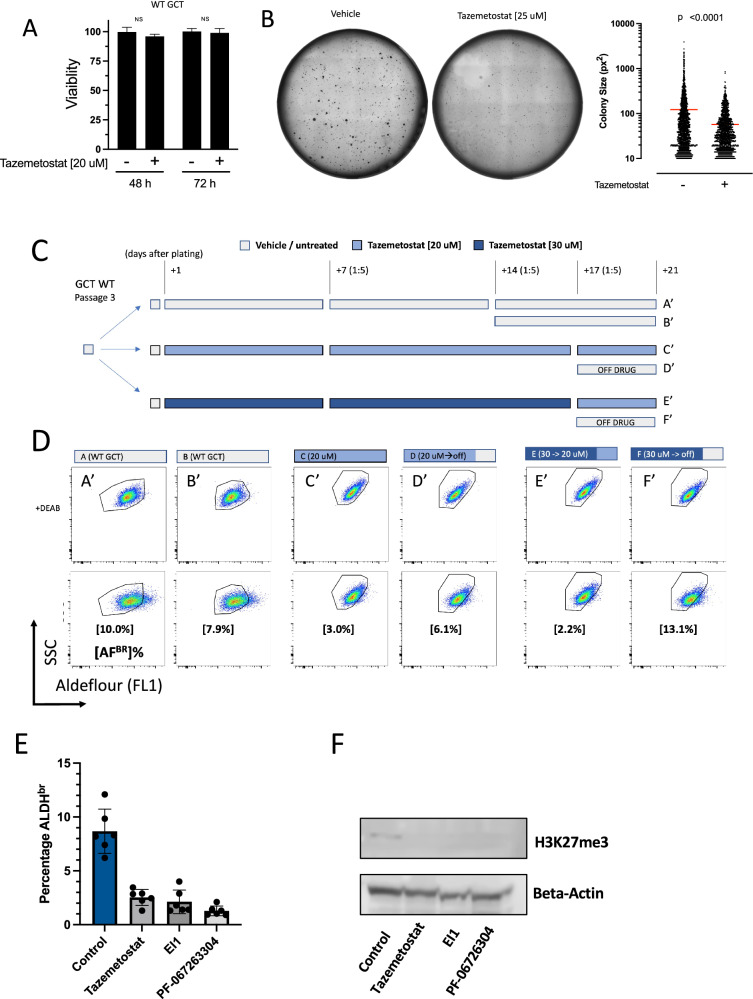
Inhibition of EZH2 with the small molecular inhibitor Tazemetostat abrogates the cancer stem cell phenotype. **A** Tazemetostat does not significantly inhibit cell viability during short term treatment. Data are the mean ± SD of three biological replicates, and are representative of three independent experiments. There is no statistically significant difference between the groups. **B** Representative soft-agar assay of WT GCT cells treated with vehicle or Tazemetostat (25 uM) (right) and quantification with imageJ (left). **C** Treatment schema of WT GCT cells subjected to long-term culture with vehicle (**A, B**) or Tazemetostat for continuous exposure (**C, E**) or on-off drug exposure (**D, F**). **D** Aldefluor assay of WT GCT cells treated with vehicle or Tazemetostat over a 3 week period. DEAB controls for each experimental arm are shown in the top row, for which the corresponding gates are directly maintained in the bottom row. The percentage of Aldefluor bright cells relative to DEAB controls is shown in brackets. **E** Continuous treatment of GCT cells with either Tazemetostat or two additional EZH2 inhibitors EI1 and PF-06726304 decreases the abundance of bright cells in the Aldefluor assay. *p < 0.001 for all three treatment groups compared to control. **F** Western blot of H3K27me3 expression in GCT cells following treatment with vehicle or the EZH2 inhibitors Tazemetostat, EI1 and PF-06726304. Beta actin is shown as a loading control.

The EZH2 pathway is upregulated and there is an increase in H3K27-me3 across CSCs from different sarcoma subtypes. We then investigated if EZH2 was required to maintain the CSC phenotype. Interestingly, we were unable to generate viable EZH2-knockout cell lines using CRISPR, suggesting the importance of this pathway in sarcoma. Therefore, we utilized a small molecule inhibitor of EZH2 enzymatic activity, Tazemetostat, which is an S-adenosyl methionine (SAM) competitive inhibitor. Tazemetostat is FDA approved for the treatment of metastatic and locally advanced epithelioid sarcoma and is well-tolerated by patients. We first evaluated the effect of Tazemetostat in GCT cells representing undifferentiated pleomorphic sarcoma, the most common complex karyotype sarcoma in adults. At 48 and 72 hours, we observed little to no effect on overall cell viability (Fig. 4A). The lack of short-term cytotoxicity in WT GCT cells was not unexpected, as we hypothesized that EZH2 specifically influences the CSC-phenotype. To investigate this possibility further, we performed soft-agar colony formation assays in cells treated with either vehicle or Tazemetostat. We found that continuous exposure to Tazemetostat significantly reduced the average size and abundance of soft-agar colonies (Fig. 4B). These data suggested that inhibition of EZH2 by Tazemetostat blocks the CSC-phenotype in soft tissue sarcoma cells. To confirm this, we analyzed the CSC population using the Aldefluor assay. Extended treatment of GCT cells with Tazemetostat results in a decrease in the percentage of Aldefluor-positive cells as compared to vehicle treatment. Removal of Tazemetostat showed that repression was transient and CSC populations rapidly returned to baseline. This data indicates that treatment with Tazemetostat specifically decreases the CSC population in soft tissue sarcomas (Fig. 4C, D). To ensure this observation is specific to EZH2 and not due to an off-target effect, we treated cells with two additional EZH2 inhibitors, EI1 and PF-06726304. Treatment with these inhibitors also resulted in a reduction in the CSC population in the Aldefluor assay (Fig. 4E). By western blot, we also confirmed that the three inhibitors decreased expression of the EZH2-target H3K27me3 in GCT cells (Fig. 4F).

### Treatment with Tazemetostat sensitizes resistant cells to Doxorubicin

**Fig. 5:**
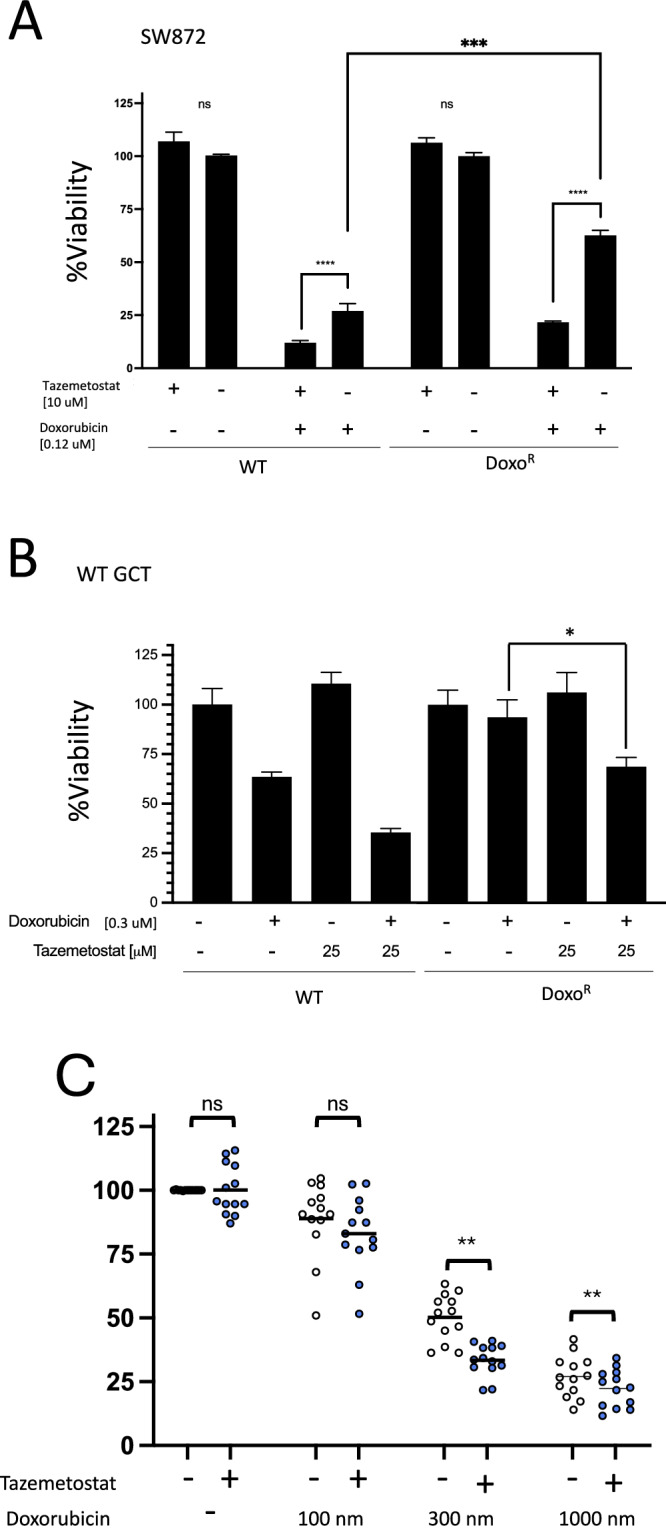
Co-treatment of soft tissue sarcoma cell lines with Tazemetostat and doxorubicin rescues treatment-acquired chemotherapeutic resistance. **A** SW872 (LPS) and derivative doxo^R^ cells treated for 96 hours with doxorubicin and Tazemetostat alone or in combination. **B** GCT (UPS) and derivative doxo^R^ cells treated for 96 hours with doxorubicin and Tazemetostat alone or in combination. Data for **A**, **B** are the mean ± SD of three biological replicates analyzed by ANOVA, * *p* < 0.05, *** *p* < 0.001 **** *p* < 0.0001. **C** Viability assays of unique, independently generated soft tissue sarcoma cell lines from oncogene-mediated forward transformation of mesenchymal stem cells in vivo treated with doxorubin at the indicated concentrations alone or in combination with 25 uM Tazemetostat. Data for A-C are the mean ± SD of three biological replicates analyzed by ANOVA, * *p* < 0.05, *** *p* < 0.001 **** *p* < 0.0001.

Given that resistance to doxorubicin was associated with an expansion of the CSC population and we found that inhibition of the epigenetic modulator EZH2 led to an ablation of the stem cell phenotype, we asked whether co-treatment of soft tissue sarcoma lines with doxorubicin and Tazemetostat would be more effective than either treatment alone. We first performed a synergy analysis in GCT cells. This is complex given that tazemetostat is not highly cytoxic and has a relatively flat dose-response curve. Viability was determined at varying doses of a single treatment or the combination. This resulted in an overall Bliss synergy score of approximately -0.2 which demonstrates an additive effect. At some dose combinations, the effects were strongly synergistic indicating that synergy may exist between these agents (Supplementary Fig. 3). To evaluate this, we performed viability assays of WT and Doxo^R^ lines with doxorubicin and Tazemetostat alone or in combination (Fig. 5). In both WT and Doxo^R^ SW872 cells, Tazemetostat had little appreciable effect on cell viability after treatment for 96 hours, consistent with earlier observations. While Tazemetostat in combination with Doxorubicin had a statistically significant effect in WT SW872 cells, this was much more pronounced in the Doxo^R^ derivative cells, completely rescuing the sensitivity of the cells to that of their WT counterparts (Fig. 5A). We observed similar findings in WT and DoxoR GCT cells (Fig. 5B). Taken together, these data support the hypothesis that EZH2 mediates the CSC phenotype in high-grade complex karyotype sarcomas and targeting of EZH2 sensitizes cells to anthracycline chemotherapy.

Having demonstrated a synergistic effect of doxorubicin and Tazemetostat in DoxoR lines, we wanted to determine if these results may be more broadly applicable. We recently developed an oncogene-driven forward genetics model in which human mesenchymal stem cells (MSCs) are transformed into high-grade soft tissue sarcomas^18^. From this, we have established a cell line panel of 13 independently derived, treatment- naïve sarcoma cell lines. These represent the undifferentiated pleomorphic sarcoma histologic subtype. We performed viability assays to determine whether these 13 unique and treatment-naïve sarcoma cell lines were uniformly or variably sensitive to doxorubicin, and whether co-treatment with Tazemetostat would potentiate these effects (Fig. 5C). We appreciated variable sensitivity to doxorubicin among the cell lines, but noted that at all concentrations of doxorubicin tested (100, 300, and 1000 nM), the addition of 25 uM Tazemetostat potentiated the effects of doxorubicin at all concentrations tested for the vast majority of the sarcoma lines. Taken together, these data strongly support the additive effect of doxorubicin and Tazemetostat in soft tissue sarcoma treatment, which can also be used to reverse acquired chemotherapeutic resistance.

## Discussion

In this study, we performed the first genetic and epigenetic analysis of CSCs across high-grade complex karyotype sarcoma subtypes to identify common therapeutic vulnerabilities. From this, we have identified EZH2 as a target to sensitize cells to anthracycline based chemotherapy. Tazemetostat, an EZH2 inhibitor is approved for treatment of SMARCB1 deficient epithelioid sarcomas^15^. This has been well tolerated by patients with a manageable side effect profile. The data provided here indicates that the use of Tazemetostat in conjunction with doxorubicin could be considered in the neoadjuvant or adjuvant setting to reduce the rate of recurrent disease. Additional approaches to overcome drug resistance such as use of tyrosine kinase inhibitors or HDAC inhibitors have been shown to be efficacious and combinations of these therapies with Tazemetostat could be considered as a chemotherapy free approach^19,20^.

The transcriptome profile of CSC to bulk population within sarcoma subtypes was different but comparing across subtypes led to few common genes. This indicates that further studies to examine each subtype individually would likely generate additional targets for investigation. Clinically, these are treated as one entity therefore, we focused on identifying a shared gene signature. Evaluating across sarcomas, this study has demonstrated the importance of EZH2 in sarcoma CSCs and established EZH2 as a potential therapeutic target. Yet, the role of EZH2 in sarcoma CSC pathophysiology is not known and warrants further investigation. Sarcoma CSCs have active developmental pathways such as nanog, hedgehog, and hippo signaling. The regulatory overlap of EZH2 with these pathways could give additional insights into sarcoma CSC biology. In other tumor types, EZH2 has been shown to regulate key downstream targets to promote and expand the CSC population^14,21,22^. It is possible that the mechanisms by which EZH2 promotes the CSC population are similar but, given that EZH2 has specific cell type and context dependent functions, further investigation is needed.

One limitation of this study is that CSC markers have not been well characterized across sarcoma subtypes. Markers such as CD133 and CD117 have been identified in specific subtypes, but, expansion of these more broadly to additional subtypes has shown a lack of specificity for the stem cell population^23–26^. Additional subtype specific markers not been investigated across the disease spectrum. Therefore, in this study, we focused on Aldefluor positivity because this has been shown to identify a CSC population across tumor types, including multiple subtypes of sarcoma such as rhabdomyosarcoma and fibrosarcoma^27–29^. It is possible that Aldefluor may not identify all of the stem cells in each subtype. Further characterization of CSCs across this heterogeneous disease would allow for more detailed future studies.

Sarcomas are treatment resistant with high rates of recurrence and metastasis presumably due to the inability to eradicate the cancer stem population. This raises the possibility that targeting of this population could improve patient outcomes. In soft tissue sarcomas, the CSC population has not been well defined or extensively evaluated across sarcoma subtypes. The findings of this study create a foundation for further translational studies aimed at improving outcomes for patients with high-grade sarcomas.

## Methods

### Cell culture and Western Blot

SW872 cells (HTB-92) were purchased from American Type Culture Collection (ATCC, Manassas, VA) cultured in L-15 media supplemented with 10% FBS (Genesee Scientific, Morrisville, NC) in 0.1% CO_2_ atmosphere. GCT cells (TIB-223) were purchased from ATCC and grown in McCoys media supplemented with 10% FBS. All cells were cultured with antibiotic (pen/strep). SK-LMS-1 leiomyosarcoma cells were purchased from, ATCC (HTB-88). RH1 (755483-174-R-J1-PDC) non-alveolar rhabdomyosarcoma sample is a patient derived tumor cell culture from the NCI Patient-Derived Models Repository (PDMR, Bethesda, MD). Cells were cultured as described by the NIH protocol using DMEM/F12 + Y compound. The myxofibrosarcoma sample is a PDX (918122-036-R) from PDMR. These were implanted into Non-SCID gamma (NSG) mice (Jackson Labs, Sacramento, CA) and once tumors reached 1 mm they were harvested, digested in collagenase/dispase (Millipore Sigma, Burlington, MA) overnight to create a single cell suspension. Doxurubicin was purchased from Sigma and diluted in DMSO. Selection was performed by serial culture in concentration ranges from 0.03 uM initially, increasing by approximately 3 fold per selection step over the course of 3 months. Cells were passaged at dilutions of 1:5-1:10 every 2-3 days. Tazemetostat, EI1, and PF-06726304 were obtained from Selleck Chemicals (Houston, TX). For western blot, H3K27me3 antibody C36B11 (Cell Signaling Boston, MA) was used in addition to beta actin (Cell signaling) as a loading control.

### Aldefluor assay

Cell culture populations representing cancer stem cells were identified using the Aldefluor assay (Stemcell Technologies, Cambridge, MA) according to the manufactures protocol, and optimized per cell line relative to reagent incubation duration. Assays were scaled using replicates as needed to achieve sufficient cell numbers for sorting.

### Flow Cytometry / Cell sorting

Flow cytometry was performed at the UC Davis Core Facility using BD Canto or Fortessa flow cytometers. Sorting was performed until sterile conditions using a BD Aria II sorter. Sorted cells were collected in respective serum-supplemented serum. Data was analyzed by FloJo software.

### Cell viability assays

Cell viability assays were performed using CellTiter Glo reagent (Promega, Madison, WI) according to the manufactuers recommended protocol. Cells were plated at a density of 2,000-10,000 cells per well in opaque 96-well white plates, cultured overnight, and then treated with serial dilutions of doxorubicin. Cells were then grown for the indicated time period, then assayed by Cell Glo-Titer. Luminescence was measured with a GloMax 96 luminometer (Promega).

### Soft agar colony formation assays

Soft agar colony formation assays were performed in 24 well plates. Stock solutions of 2x noble agar (1.2 and and 0.6%) were prepared, sterilized, and stored at 4 C until needed. On the day of preparation, stock solutions were warmed and the base layer was prepared by combining with a 2X solution of DMEM/F12, FBS, pen/strep (Genesee) and kept at 42 C until used. The base layer (300 uL, 0.6% final agar concentration) was placed with pipette and allowed to solidify at room temperature. Cells were collected, counted, mixed as a 2X stock and combined with agar (0.3% final agar concentration, 10,000 cells/well), allowed to cool to room temp, and then covered with 100 uL of media. For quantification, wells were imaged in entirety using an EVOS M5000 microscope in a grid, stitched together using Image J software, thresholded, and then counted using the analyze particle function^30^.

### TCGA and Synergy Analysis

Survival analysis linked to sarcoma-specific tumors was performed using an online web tool (Oncolnc.org). Synergy analysis of doxorubicin and tazemetostat was performed using synergy finder (synergyfinder.fimm.fi) for Bliss evaluation.

### Xenografts

Xenograft tumors of SW872 and GCT cells (and their doxorubicin-resistant derivatives) were established in immunocompromised NSG mice (Jackson Labs Cat#005557) after injection of a 1:1 mixture of cells in PBS and Matrigel (1×10^6 cells/injection). All animal work was conducted at UC Davis and approved by the institutional animal care and use committee. Tumor growth was checked weekly and collected once palpable. Tumors were placed in formalin and processed for H&E staining by the pathology core at UC Davis.

### RNA Sequencing

Indexed, stranded mRNA-seq libraries were prepared from total RNA using the KAPA mRNA HyperPrep Kit (Roche) according to the manufacturer’s standard protocol for mRNA capture, fragmentation, random-primed first strand synthesis, second strand synthesis with dUTP marking, A-tailing, adaptor ligation, and library amplification. Libraries were pooled and multiplex sequenced on an Illumina NovaSeq 6000 System (150-bp, paired-end, >30 × 10^6 reads per sample). De-multiplexed raw sequence reads (FASTQ format) were mapped to the reference human transcriptome index (GRCh38/hg38, GENCODE release 36) and quantified with *Salmon*^31^. Gene-level read counts were then imported with *tximport*^32^. Differential expression analysis was performed with DESeq2^33^ and results were filtered for statistical significance (i.e., adjusted p < 0.05). Principal components analysis (PCA), hierarchical clustering, and Gene Set Enrichment Analysis (GSEA) (4,5) were performed on normalized read count data. For visualization of RNA-seq coverage tracks (bigWig) in the Integrative Genomics Viewer (IGV)^34^, read alignment was performed using the Spliced Transcripts Alignment to a Reference (STAR) ultrafast splice-aware aligner^35^ followed by generation of depth/control-normalized bigWig files with deepTools2^36^ using bins per million mapped reads (BPM) read coverage normalization.

### ATAC Sequencing

Sorted ALDH bright and dim cells (100,000) were used as input for library preparation. The protocol was followed to produce Illumina compatible libraries using the ATAC-seq kit from Active Motif. Briefly, cells were lysed and underwent tagmentation with Tn5 transposase and sequencing adapters. Illumina libraries sequenced using a NovaSeq 6000 (2 x 150 bp, 45 ×10^6 reads per sample). Raw ATAC-seq data (FASTQ format) was analyzed using the nf-core/atac-seq pipeline (v1.2.1; https://nf-co.re/atacseq/1.2.1)^37^. This performed sequence read quality control analyses with FastQC^38^, adapter trimming, mapping to the reference human genome assembly (GRCh38/hg38) with Burrows-Wheeler alignment^39^, and post-alignment data cleanup steps, such as duplicate marking (picard) and read filtering. Normalized bigWig files were created and passed to deepTools^36^ to generate gene-body metaprofiles and calculate genome-wide enrichment. Peaks calling was performed with the Model-based Analysis of ChIP-Seq (MACS2) algorithm^40^ followed by annotation with HOMER (Hypergeometric Optimization of Motif EnRichment)^41^, and creation of consensus peaksets from across all samples with BEDTools utilities.

## Supplementary information

Supplemental Figures 1-3

## Data Availability

RNA sequencing and ATAC-seq data are publicly available at the Gene Expression Omnibus repository.
