# Supplementary material for: Genetic and epigenetic characterization of sarcoma stem cells across subtypes identifies EZH2 as a therapeutic target
Source: NPJ Precis Oncol. 2025 Jan 9;9:7. doi: 10.1038/s41698-024-00776-7 (PMC11717953; doi:10.1038/s41698-024-00776-7)
Supplement: Supplementary file 1 — Supplemental Figures 1-3 [file 41698_2024_776_MOESM1_ESM.pdf]

Supplementary Figure 1

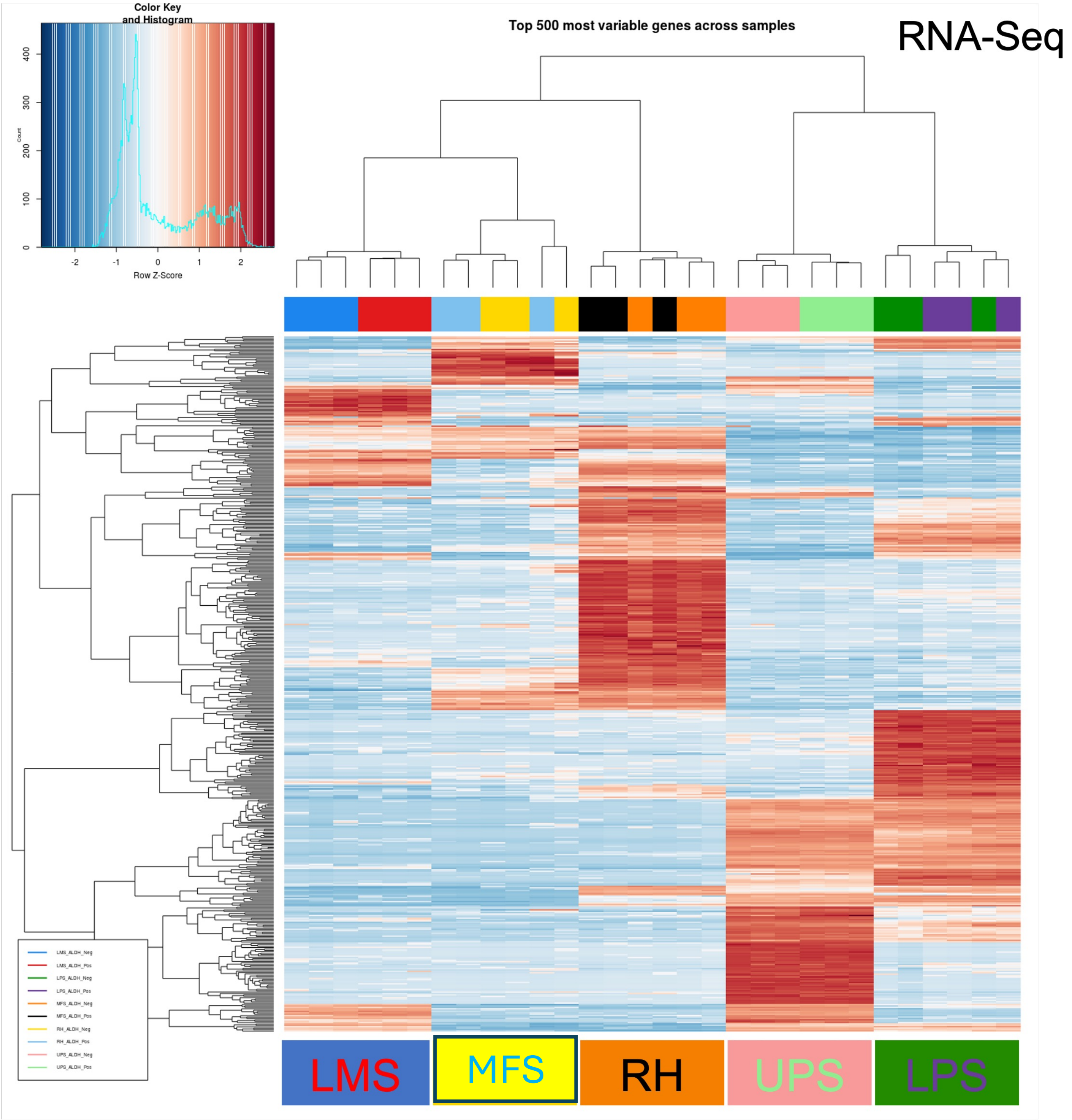

Supplementary Figure 1. Heatmap of the top 500 differentially regulated genes on RNA-seq analysis of Aldefluor bright and dim sorted cells among five soft tissue sarcoma cell lines showin in Figure 2. LMS=leiomyosarcoma (SKLMS1), RH=rhabdomyosarcoma, MFS=myxofibrosarcoma, UPS=undifferentiated pleomorphic sarcoma (GCT), LPS=dedifferentiated liposarcoma (SW872). Within each column set, the font color corresponds to "bright" sorted samples and background color corresponds to "dim" sorted samples for the color bars at the top of the diagram.

Supplementary Figure 2

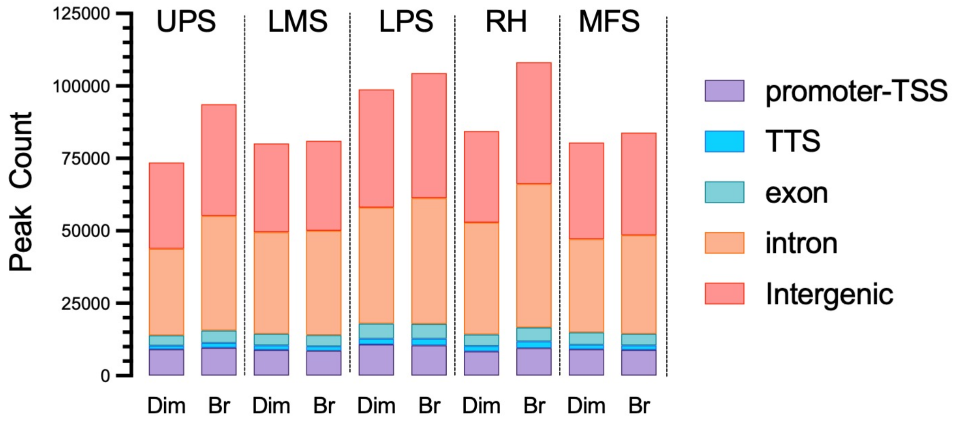

Supplementary Figure 2. Distribution of ATAC-seq peaks. Reads were annotated according to genomic location (promotor transcription start site, transcription termination site, exon, intron, and intergenic) and plotted according to bright or dim Aldefluor activity for the five sarcoma cell lines

Supplementary Figure 3

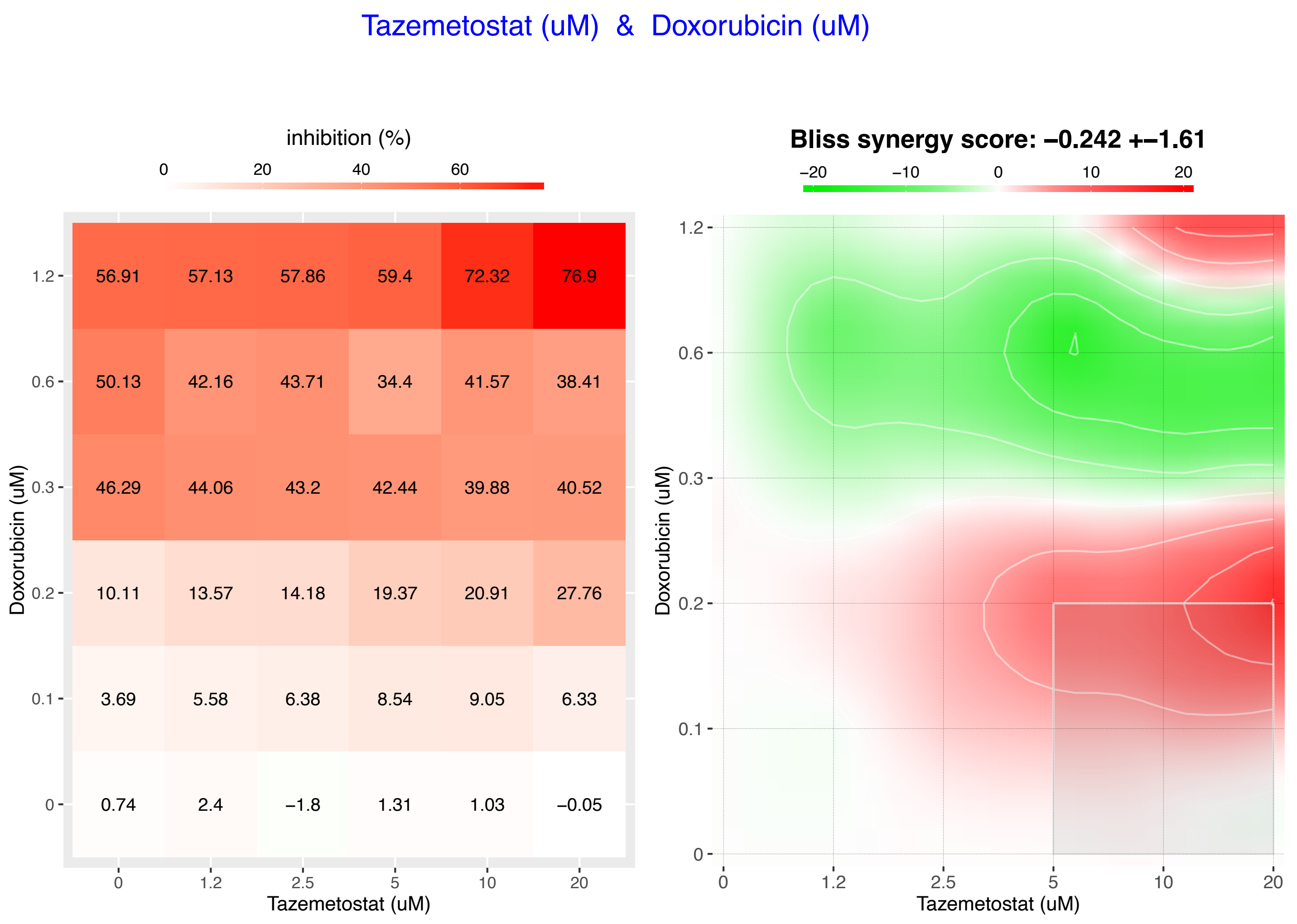

Supplementary Figure 3. Synergy analysis in GCT cells. Viability was determined by cell-titer glo assay at varying doses of single treatment or combinations at the indicated doses. Data was analyzed using syngeryfinder as described in the methods.
